# Cured meat, vegetables, and bean-curd foods in relation to childhood acute leukemia risk: A population based case-control study

**DOI:** 10.1186/1471-2407-9-15

**Published:** 2009-01-13

**Authors:** Chen-yu Liu, Yi-Hsiang Hsu, Ming-Tsang Wu, Pi-Chen Pan, Chi-Kung Ho, Li Su, Xin Xu, Yi Li, David C Christiani, the Kaohsiung Leukemia Research Group

**Affiliations:** 1 Environmental and Occupational Medicine and Epidemiology Program, Department of Environmental Health, Harvard School of Public Health, Boston, MA, USA; 2 Institute for Aging Research, Hebrew Rehabilitation Center, Harvard Medical School, Boston, MA, USA; 3 Molecular and Integrative Physiological Sciences Program, Department of Environmental Health, Harvard School of Public Health, Boston, MA, USA; 4 Department of Family Medicine and Graduate Institute of Occupational Safety & Health, Kaohsiung Medical University, Kaohsiung, Taiwan; 5 Center of Excellence for Environmental Medicine, Kaohsiung Medical University, Kaohsiung, Taiwan; 6 Department of Nursing, Yuh-Ing Junior College of Health Care and Management, Kaohsiung, Taiwan; 7 School of Public Health, Kaohsiung Medical University, Kaohsiung, Taiwan; 8 Department of Biostatistics and Computational Biology, Dana-Farber Cancer Institute, Boston, MA, USA; 9 Department of Biostatistics, Harvard School of Public Health, Boston, MA, USA; 10 Pulmonary and Critical Care Unit, Department of Medicine, Massachusetts General Hospital, Harvard Medical School, Boston, MA, USA

## Abstract

**Background:**

Consumption of cured/smoked meat and fish leads to the formation of carcinogenic *N-*nitroso compounds in the acidic stomach. This study investigated whether consumed cured/smoked meat and fish, the major dietary resource for exposure to nitrites and nitrosamines, is associated with childhood acute leukemia.

**Methods:**

A population-based case-control study of Han Chinese between 2 and 20 years old was conducted in southern Taiwan. 145 acute leukemia cases and 370 age- and sex-matched controls were recruited between 1997 and 2005. Dietary data were obtained from a questionnaire. Multiple logistic regression models were used in data analyses.

**Results:**

Consumption of cured/smoked meat and fish more than once a week was associated with an increased risk of acute leukemia (OR = 1.74; 95% CI: 1.15–2.64). Conversely, higher intake of vegetables (OR = 0.55; 95% CI: 0.37–0.83) and bean-curd (OR = 0.55; 95% CI: 0.34–0.89) was associated with a reduced risk. No statistically significant association was observed between leukemia risk and the consumption of pickled vegetables, fruits, and tea.

**Conclusion:**

Dietary exposure to cured/smoked meat and fish may be associated with leukemia risk through their contents of nitrites and nitrosamines among children and adolescents, and intake of vegetables and soy-bean curd may be protective.

## Background

Leukemia is the most frequent type of childhood cancer [1]. Acute lymphoblastic leukemia (ALL), representing 80% of diagnoses, is the main subtype of childhood leukemia followed by acute myeloid leukemia (AML) and the chronic subtypes of leukemia [1]. Lower incidence of childhood leukemia has been documented among Chinese, Japanese, and Koreans than among Caucasians [2]. Several risk factors are thought to play a role in the hematopoietic carcinogenesis. These factors include environmental factors (e.g., benzene, ionizing radiation, nonionizing electromagnetic fields, pesticides, occupations, and parental occupations), medical events (e.g., radiation therapy, and chemotherapy agents), and familial and genetic factors. However, not all leukemia cases are explainable by these factors [3-5].

Nutrition has been previously implicated in playing a complex role in cancer etiology. Carcinogen exposure may occur through preservation methods and high temperature cooking [6]. Chinese-style salted fish is characterized as a Group 1 carcinogen by the International Agency for Research on Cancer [7]. Consumption of cured/smoked meat and fish, which contains *N-*nitroso precursors, leads to the formation of carcinogenic *N-*nitroso compounds in the acidic stomach [8]. Antioxidants, such as vitamin C, E, flavones and flavanones in fresh fruits, vegetables, green tea, and soybeans were found to block the nitrosation reaction [9-11]. Epidemiologic studies have suggested that increased consumption of cured meat is associated with a higher risk of colorectal cancer and stomach cancer [12]; while consumption of fresh fruits and vegetables is associated with a decreased risk of breast, colon, lung, pancreas, bladder, larynx, stomach, esophageal, and oral cancers [13-16].

Compared to the solid tumors, few studies have reported the effects of diet in relation to childhood leukemia and have inconsistent results. In addition, all of them were conducted in Western countries [17-19]. To assess children's diet and the risk of childhood leukemia, we performed an analysis in a population based case-control study in a Han Chinese population.

## Methods

### Study population

This population-based case-control study was conducted in metropolitan Kaohsiung, southern Taiwan. Details of the study area as well as the cases and controls recruitment criterion were described previously [20,21]. Briefly, cases and controls were selected from the non-agricultural areas in Kaohsiung to avoid potential confounding effects of pesticides. Incident leukemia cases were identified on the basis of International Classification of Disease 9^th ^Revision (ICD-9) criteria, codes 204–208. Cases were histologically confirmed to have an incident, primary diagnosis of leukemia (International Classification of Disease 9^th ^Revision criteria, codes 204–208), less than 20 years old, diagnosed between November 1997 and December 2005 and residents of the study area at the time of enrollment. Patients with secondary or recurrent tumors were excluded. Cases recruitment was performed through two systems including the rapid case ascertainment system from the large referral hospitals in the study area and computer files abstracted from records of the mandatory national health insurance system operated by the Department of Health of Kaohsiung, Taiwan. The former was set up by the Kaohsiung Medical University Hospital to obtain information regarding newly diagnosed cases from the large referral hospitals in the study area (Kaohsiung Medical University Hospital, Kaohsiung Chang-Gung Memorial Hospital, and Kaohsiung Veterans General Hospital). All citizens who have established a registered domicile for at least 4 months in Taiwan are covered by a National Medical Insurance System and have access to these referral hospitals. With these two case ascertainment combined, all of the cases occurring in metropolitan Kaohsiung have been identified. All case diagnoses were confirmed by an experienced pathologist in the Kaohsiung Medical University Hospital.

After each incident case was identified, matched controls were randomly selected using a personal identification number assigned by the Household Registration Office from the population registry data of the chosen study area. This number assignment is independent of current residence and would not bias the control selection by residence. Individuals with any known malignancy were excluded from the controls of the study. Cases and controls were matched on age (± 1 year) and gender using a 1:3 matching ratio.

Totally 190 eligible cases and 842 eligible controls were identified. Among them, 179 cases (94%) and 475 controls (56%) agreed to participants. The reasons for nonparticipation for the eligible cases included refusal (2 percent); could not be contacted (1 percent); missing address information (2 percent); and parents were divorced or widowed (1 percent). Reasons for nonparticipation for the eligible controls included refusal (22 percent); could not be contacted (14 percent); missing address information (6 percent); and parents were divorced or widowed (2 percent). Therefore, not all of the cases had 3 matched controls. The differences between participants and non-participants by residence are not statistically significant [21].

The study protocol was approved by the Institutional Review Boards of the Harvard School of Public Health and Kaohsiung Medical University. All study subjects ≥ 18 years of age consented; or their parents (if < 18 years of age) assented to participate in the study.

### Questionnaires

A trained interviewer conducted an in-person interview immediately after participants were recruited. Follow-up phone interviews were administered occasionally if required. Either the subject's biologic mother or the subject himself/herself completed the questionnaire. A modified questionnaire originally developed by Children's Cancer Group and the Pediatric Oncology Group Study [22] was used to obtain information on the subjects' socio-demographic characteristics, medical history, residential history up to 2 years prior to birth, occupational history (if the subject was ≥ 16 years of age), cigarette smoking, alcohol consumption, diet, and exposure to various hazardous agents. The questionnaire was translated from English into traditional Chinese, and back-translated independently to assure accuracy. Some modifications were done for better cultural adaptation. For example, Chinese style sausage, salted fish, and dried salted duck were added in the food group of cured/smoked meat or fish. A portion of the habitual diet questionnaire concentrated on consumption of the following foods/food groups: fruits, vegetables, bean-curd foods (to-fu, dried to-fu, to-fu pudding, etc), tea (black tea, green tea, and oolong tea), alcohol-containing beverages (beer, spirits, and wine), cured/smoked meat or fish (Chinese style sausage, salted fish, preserved meat, bacon, ham, hot dog, dried salted duck), and pickled vegetables (pickled Chinese cabbage, pickled potherb mustard, salted cabbage, etc). Interviewers asked the cases to estimate their usual intake of these food groups prior to diagnosis. Controls were asked to estimate their usual intake before the recruitment. For each food/food group, participants reported their frequency of intake. The usual dietary intakes listed in the questionnaire were in frequency categories (as the original food groups in Table 1). The frequency categories for the responses were never, > = 1/week for more than 6 months (ever) for tea and alcohol-containing beverages; < 1/week, 1/week, 2–3/week, 4–6/week, few times/day for fruits and vegetables; never, few times/year, < 4/month, 1–2/week, 3–4/week, > = 5/week for bean-curd foods, cured/smoked meat and fish, and pickled vegetables. Portion size was not measured.

**Table 1:** Definition of the regrouped food frequency categories for data analysis based on original categories obtained from the child's diet questionnaire

Food group	New categries for data analysis	
	
	Rare or occasional consumption	Frequent consumption
Cured meat/fish	Never; few times/year;< 4/month	1–2/week; 3–4/week; > = 5/week
Pickled vegetables	Never; few times/year	< 4/month; 1–2/week; 3–4/week; > = 5/week
Bean-curd foods	Never; few times/year	< 4/month; 1–2/week; 3–4/week; > = 5/week
Vegetables	< 1/week; 1/week; 2–3/week; 5–6/week	few times/day
Fruits	< 1/week; 1/week; 2–3/week; 5–6/week	few times/day

A similar questionnaire for the mother was used with additional questions added to obtain information on maternal reproductive history, alcohol-containing beverages and supplement use (vitamins and iron supplements) and/or medication usage 21 months before delivery to the study subject's date of birth or the date breast-feeding stopped.

### Statistical analyses

In the analyses, the frequency categories of food consumption on the questionnaire were collapsed into simpler categories for sufficient numbers of cases and controls for each category in each food/food group. The cut-points for the regrouped categories were based on the median consumption frequencies in the controls. New categories were used to test if the childhood leukemia risk is associated with frequent cured/smoked meat or fish and pickled vegetables consumption (above median) and the rare and occasional fruits, vegetables, and bean-curd foods consumption (below median). The regrouped food frequency categories are summarized in table 1.

The data was analyzed using the SAS^® ^software version 9.1 (SAS Institute, Cary, North Carolina). Among the identified 179 acute leukemia cases (136 ALL and 43 AML) and 475 controls, we restricted our analysis to Han Chinese participants to reduce confounding by ethnicity (158 cases and 404 controls). In addition, since it is less likely for infants to have habitual cured or pickled foods intake and the potential different risk factors for infants who develop leukemia [23], cases and their respective controls under 2 years of age were excluded. 145 acute leukemia cases (112 ALL and 33 AML) and 370 matched controls are in the analyses. Selected principal characteristics were evaluated by χ^2^, Fisher exact, and *t *tests, as appropriate.

Analyses of the frequency of consumption of each food/food group and risk of leukemia were performed using logistic regression models with adjustments made for age, sex, maternal age, birth weight, breastfeeding, parental education levels, parental and subjects' smoking history, maternal vitamins and iron supplements intake status, and all the other food items. As no confounding was seen by these variables alone or together on the associations of interest, the results presented are only adjusted for the matching factors including age and sex. A Wald's test based on the original ordinal dietary variables obtained from the child's diet questionnaire was used to test for trend. Stratified analyses were performed to estimate the combined dietary effects.

All reported p values are from two-sided tests. An adjusted p-value less than 0.05 was considered to be statistically significant.

## Results

### Demographics

The distributions of selected characteristics among study subjects are shown in table 2. There were no significant differences between the acute leukemia cases and controls with respect to maternal age, birth weight, breastfeeding, maternal, paternal, and subjects' smoking history, and parental education levels. None of the study subjects reported alcohol consumption. Neither the offspring's nor the mothers' employment was in petrochemical-related occupations.

**Table 2:** Principal characteristics of childhood acute leukemia cases and controls age between 2 and 20 years

	ALL		AML	
		
Variable^a^	Case (n = 112)	Control (n = 279)	Case (n = 33)	Control (n = 91)
**Age (years) (median (SD))**	6.9 (5.6)	7.8 (5.5)	14.6 (4.9)	14.8 (4.9)
**Sex**				
**Male (%)**	76 (67.9)	185 (66.3)	18 (54.5)	53 (58.2)
**Female (%)**	36 (32.1)	94 (33.7)	15 (45.5)	38 (41.8)
**Maternal age (years)**				
**< 25 (%)**	27 (24.5)	73 (26.3)	6 (18.2)	21 (23.1)
**25–34 (%)**	74 (67.3)	185 (66.5)	27 (81.8)	68 (74.7)
**≧ 35 (%)**	9 (8.2)	20 (7.2)	0 (0.0)	2 (2.2)
**Birth weight (g)**				
**< 3000 (%)**	27 (24.1)	72 (25.8)	11 (33.3)	25 (27.5)
**3000–3499 (%)**	48 (42.9)	134 (48.0)	10 (30.3)	42 (46.2)
**≧ 3500 (%)**	37 (33.0)	73 (26.2)	12 (36.4)	24 (26.4)
**Breastfeeding**				
**Never (%)**	70 (62.5)	157 (56.3)	25 (75.8)	59 (64.8)
**Ever (%)**	41 (36.6)	121 (43.3)	8 (24.2)	32 (35.2)
**Unknown (%)**	1 (0.9)	1 (0.4)	0 (0.00)	0 (0.0)
**Maternal smoking during pregnancy**				
**Yes (%)**	1 (0.9)	4 (1.4)	1 (3.0)	0 (0.0)
**No (%)**	111 (99.1)	275 (98.6)	32 (97.0)	91 (100.0)
**Paternal smoking during pregnancy**				
**Yes (%)**	59 (53.6)	152 (54.5)	20 (60.6)	55 (50.4)
**No (%)**	51 (46.4)	127 (45.5)	13 (39.4)	36 (39.6)
**Subjects smoking before diagnosis**				
**Yes (%)**	1 (0.9)	6 (2.2)	0 (0)	1 (1.1)
**No (%)**	111 (99.1)	273 (97.8)	33 (100)	90 (98.9)
**Parental education levels^b^**				
**Not junior high school graduated (%)**	5 (4.5)	15 (5.4)	0 (0.00)	10 (11.0)
**Junior high school graduated (%)**	9 (8.0)	35 (12.5)	7 (21.2)	13 (14.3)
**High school graduated (%)**	55 (49.1)	114 (40.9)	13 (39.4)	41 (45.1)
**Four-year college/university and above (%)**	43 (38.4)	115 (41.2)	13 (39.4)	27 (29.7)

^a^Number and percent of group (%) or (median(SD)). Percents are rounded

^b^Highest education level of either parent

### Dietary effects

Compared to the consumption frequency from the nationwide survey using 24-hour recalls, the frequency of each food consumption among the controls in our study was similarly distributed to the nationwide survey of the same age groups [24] (data not shown). Therefore, our control selection is comparable to the general population in Southern Taiwan, from which all cases were selected. To increase statistical power, we combined ALL and AML together. As shown in table 3, twenty-four percent of the controls consumed cured/smoked meat/fish frequently. Frequent consumption of cured/smoked meat and fish, consumption more than a few times a week, was associated with an increased risk of acute leukemia, with an odds ratio of 1.74 (95% CI: 1.15–2.64; *p*_trend _= 0.03). Consumption of bean-curd foods was significantly associated with a reduced risk of the childhood acute leukemia. Eighty-five percent of the controls consumed bean-curd foods frequently. Compared to the rare or occasional consumption category, the odds ratio was 0.55 (95% CI, 0.34–0.89; *p*_trend _> 0.05) for frequent consumption in relation to childhood acute leukemia risk. Seventy-four percent of the controls reported frequently consumed vegetables. Frequent consumption of vegetables was associated with a reduced risk of childhood acute leukemia (OR = 0.55; 95% CI: 0.37–0.83; *p*_trend _= 0.001). Tea, fruits, and pickled vegetables consumption did not show significant associations on childhood acute leukemia.

**Table 3:** Frequencies and adjusted odds ratios of diet effects on acute leukemia risk

	2–5 years old			2–20 years old		
	
	Cases	Controls		Cases	Controls	
						
Consumption of food	(n = 50)	(n = 118)	OR (95% CI)^a^	(n = 145)	(n = 370)	OR (95% CI)^a^
**Cured meat/fish**						
Rare or occasional	34	90	1.00	94	282	1.00
Frequent	16	28	1.52 (0.75–3.09)	51	88	**1.74 (1.15–2.64)^c^**
**Pickled vegetables**						
Rare or occasional	44	103	1.00	124	322	1.00
Frequent	6	15	1.07 (0.41–2.79)	20	48	1.10 (0.62–1.93)
**Bean-curd food**						
Rare or occasional	17	21	1.00	34	53	1.00
Frequent	33	97	**0.47 (0.22–0.99)^b^**	111	317	**0.55 (0.34–0.89)^b^**
**Vegetables**						
Rare or occasional	27	39	1.00	57	97	1.00
Frequent	23	79	**0.44 (0.22–0.85)^b^**	88	273	**0.55 (0.37–0.83)^c^**
**Fruits**						
Rare or occasional	29	46	1.00	75	178	1.00
Frequent	21	72	0.53 (0.28–1.01)	70	192	0.86 (0.58–1.26)
**Tea**						
No	34	80	1.00	100	237	1.00
Yes	16	38	0.98 (0.49–1.95)	45	133	0.80 (0.53–1.22)

^a^Odds ratios and 95% confidence intervals derived from logistic regression adjusted for age and sex

^b^p < 0.05; ^c^p < 0.01

Analyses stratified by ALL and AML cases and their matched controls yielded similar results. Frequent consumption of cured/smoked meat and fish was associated with an increase risk of ALL (OR = 1.49; 95% CI: 0.92–2.42) and AML (OR = 2.76; 95% CI: 1.19–6.41). Conversely, higher intake of bean-curd (OR = 0.54; 95% CI: 0.31–0.95) and vegetables (OR = 0.49; 95% CI: 0.31–0.78) was associated with a reduced ALL risk. The associations between AML and the consumptions of bean-curd and vegetables are not statistically significant.

To estimate the dietary effect in early childhood, the models were restricted to acute leukemia cases diagnosed between the ages of 2 and 5 years and their matched controls. Significant protective associations were observed among children with higher intake of bean-curd foods and vegetables. Frequent consumption of cured/smoked meat and fish, though the odds ratio was no longer significant, was associated with an increased risk of acute leukemia (Table 3).

#### Other dietary effects

We assessed carefully the socioeconomic status and other principal characteristics that may influence participants' diet and showed no significant differences between cases and controls (Table 2). Adjustment for all these characteristics in models did not affect the observed associations. We did not find significant associations of childhood acute leukemia risk with maternal vitamin, iron supplements, and alcohol-containing beverage intake during pregnancy (data not shown)

#### Combined dietary effects

Participants consume vegetables frequently may consume less cured meat/fish, but more bean-cured food. To estimate the independent effects of these three food consumption on the risk of childhood acute leukemia, we performed additional analyses to estimate the combined dietary effects (Table 4). Compared to participants with both rare cured meat/fish and vegetables, subjects with frequent consumption of cured meat/fish have a 2.7-time higher risk of having leukemia. This effect is independent from vegetable consumption. Compared to participants with both rare cured meat/fish and vegetables, subjects with frequent consumption of vegetable have a 40% reduced risk of having leukemia. This effect is independent from cured meat/fish consumption. However, since vegetables and bean-curd food usually mixed together in Chinese food, it's difficult to distinguish the effects contributed to the risk of childhood leukemia. We also performed analyses with adjustment for all the other food items and found no confounding effects for the observed associations (the ORs changed < 10%).

**Table 4:** Relationships between combined dietary factors and acute leukemia risk

		Cases	Controls	
Consumption of food		(n = 145)	(n = 370)	OR (95% CI)^a^
**Cured meat/fish**	**Vegetables**			
Rare or occasional	Rare or occasional	38	82	1.00
Frequent	Rare or occasional	19	15	**2.73 (1.26–5.96)^c^**
Rare or occasional	Frequent	56	200	**0.61 (0.37–0.99)^b^**
Frequent	Frequent	32	73	0.95 (0.54–1.67)
**Cured meat/fish**	**Bean-curd food**			
Rare or occasional	Rare or occasional	24	48	1.00
Frequent	Rare or occasional	10	5	**4.01 (1.23–13.05)^b^**
Rare or occasional	Frequent	70	234	0.60 (0.34–1.05)
Frequent	Frequent	41	83	0.99 (0.53–1.83)
**Vegetables**	**Bean-curd food**			
Rare or occasional	Rare or occasional	19	25	1.00
Frequent	Rare or occasional	15	28	0.70 (0.30–1.68)
Rare or occasional	Frequent	38	72	0.69 (0.34–1.42)
Frequent	Frequent	73	245	**0.39 (0.20–0.75)^c^**

^a^Odds ratios and 95% confidence intervals derived from logistic regression adjusted for age and sex

^b^p < 0.05; ^c^p < 0.01

## Discussion

In this study, we found that the consumption of cured/smoked meat and fish is associated with an increased risk of acute leukemia; while bean-curd foods and vegetables is associated with a reduced risk of childhood acute leukemia. We found no apparent associations for consumption of pickled vegetables, tea, fruits and risk of acute leukemia. To our knowledge, this is the first study that addressed the association of child's diet and the leukemia risk in a Chinese population.

Our study confirmed previous findings conducted in California, USA by Peters et al [18]. In their study, cured/smoked meat consumption was associated with a higher risk of developing childhood leukemia (OR = 9.5; 95% CI: 1.6–57.6) for child's consumption of 12 or more hot dogs a month versus none during the reference period. Consumption of cured/smoked meat and fish, which contain *N*-nitroso precursors, could lead to the formation of carcinogenic *N*-nitroso compound in the acidic stomach [8]. However, no significant association between cured/smoked meat consumption and leukemia risk was reported in two other studies [17,19]. In these two studies, a smaller proportion of children reported frequent cured/smoked meat consumption [17,19]. In our study, 24% of the controls and 35% of the acute leukemia cases consumed cured/smoked meat and fish more than once a week. In addition, differences in the food preparation methods among studies and over time may result in varied amounts of contaminants.

The traditional Chinese diet is characterized by a high intake of bean curd foods and vegetables, but a low intake of fried or grilled foods. Our study found that, compared to subjects with rare consumption of bean-curd foods and vegetables, individuals with frequent consumption have a lower risk of acute leukemia. Vegetables provide nitrosation-inhibiting antioxidants such as contain vitamin C and E, carotenoids, tocopherols, and selenium [25]. The findings of the consumption of vegetables associated with a reduced risk of childhood leukemia are consistent with previous studies [19,26,27]. Epidemiological studies have demonstrated an association between the consumption of soybean and a reduced risk for breast cancer [28,29], prostate cancer [30,31], stomach cancer [32], nasopharyngeal carcinoma [33], cardiovascular disease [34], and atherosclerosis [35]. Soy isoflavones and soy protein are mainly found in soybeans and soy foods. Many studies have focused on their antiestrogenic properties in possibly preventing hormonally mediated cancers. Consumption of soy foods may be associated with a reduced risk of non-hormonally dependent cancers as well as hormonally dependent cancers. In addition to the antiestrogenic properties, studies have reported that soy isoflavones are associated with inhibition of cancer initiation by inducing detoxifying enzymes [36,37] and its antioxidant [38] and antitumor activities, including inhibition of angiogenesis [39], topoisomerase [40], and tyrosine kinase [41]. Experimental studies have suggested that consumption of dietary antioxidants can block endogenous nitrosation [9,10]. Our study found that, among the frequent cured/smoked meat and fish consumers, the risk of the participants rarely consumed vegetables or bean-curd foods is about 3 times or higher in comparison to the participants frequently consumed vegetables or bean-curd foods (Table 4).

There are several strengths of this present study including: a) the selection of controls from a general population registry; b) an adequate sample size, considering that leukemia is a relatively rare disease and we studied a relatively small geographic area of a single ethnic group; c) comprehensive information was collected to adjust for several potential confounders/effect modifiers reported by previous studies, including maternal age, birth weight, breastfeeding, parental and subjects' smoking history, and parental education levels.

Several potential limitations should be considered when interpreting our study results. Molecular studies indicate that early childhood leukemia can originate prenatally [42-44]. Studies have shown that maternal consumption of vegetables, fruits and vitamin supplement during pregnancy may reduce the risk [23,45,46]. Maternal consumption of vegetables and fruits during pregnancy were not measured in our study. Moreover, lacking portion size and information on food preparation method could result in different levels of contaminants.

In addition, since the primary focus of the hypothesis is the effect of *N*-nitrosamines dietary exposure in relation to acute leukemia, only these specific food groups were collected in the questionnaire. Several other uncollected dietary factors may confound or modify the association of leukemia risk. For example, the cured/smoked meat and fish are often cooked or consumed with garlic (Allium sativum), which contains several anti-cancer compounds [47]. Although misclassification by dietary exposure to garlic is likely to be non-differential and may attenuate the odds ratio of cured/smoked meat and fish, we cannot rule out that our finding may be due to other unknown dietary or lifestyle factors.

Another potential problem is recall bias, where differential misclassification of dietary intake between cases and controls may overestimate the association between diet and cancer [48]. Our cases and controls were identified between 1997 and 2005. The food supply and dietary patterns may have changed during the 8-year period (1997–2005). However, the controls were matched at age and time of diagnosis with incident cases. Furthermore, the cases and controls were interviewed in the similar time points and conducted immediately after the subjects recruited to reduce potential measurement errors from recent dietary changes after diagnosis of cancer as well as misclassification from recall bias. In addition, there was no public perception at the time of data collection that child's diet is associated with leukemia risk. The effects of changes in the food supply and the transitions in dietary pattern by time should be similar among cases and controls. It is thus unlikely that the association observed was biased by time. Therefore, it is unlikely that the observed association was a result of exposure misclassification. We believe our findings justify further testing of this hypothesis with other studies.

## Conclusion

Our findings suggest that consumption of common daily Chinese diet, such as vegetables and soy-foods may be associated with a reduced risk of childhood acute leukemia; while consumption of cured/smoked meat/fish may increase the risk of childhood acute leukemia. Further studies with more detailed diet and biomarker assessments are necessary to confirm our findings and to elucidate potential mechanisms.

## Competing interests

The authors declare that they have no competing interests.

## Authors' contributions

CYL, MTW, PCP, CKH, and DC developed the design of the study. CYL, YHH, and DC did the data analysis and interpretation of data. CYL drafted the manuscript and all other authors were involved revising the manuscript critically. All authors have given final approval of the version to be published.

## Pre-publication history

The pre-publication history for this paper can be accessed here:

http://www.biomedcentral.com/1471-2407/9/15/prepub

## References

[B1] Ries LAGSM Gurney JG Linet M Tamra T Young JL Bunin GR (eds) Cancer Incidence and Survival among Children and Adolescents: United States SEER Program 1975-1995, National Cancer Institute, SEER Program 1999 NIH Pub. Bethesda, MD 17 34

[B2] Miller BAKL Bernstein L Young JL Jr Swanson GM West D Key CR Liff JM Glover CS Alexander GA (eds) Racial/Ethnic Patterns of Cancer in the United States 1988–1992 1996 National Cancer Institute NIH Pub No 96-4104

[B3] Brandt L Environmental factors and leukaemia Med Oncol Tumor Pharmacother 1985 2 1 7 10 3903369 10.1007/BF02934774

[B4] Linet MS CRTlISD Fraumeni JF (eds) Cancer Epidemiology and Prevention 1996 2 841 892

[B5] Zeeb H Blettner M Adult leukaemia: what is the role of currently known risk factors? Radiat Environ Biophys 1998 36 4 217 228 10.1007/s004110050075 9523337

[B6] Ferguson LR Natural and human-made mutagens and carcinogens in the human diet Toxicology 2002 181–182 79 82 10.1016/S0300-483X(02)00258-5 12505288

[B7] IARCmonographs on the evaluation of carcinogenic risks to humans. Suppl 7. Overall evaluations of carcinogenicity: an updating of IARC monographs volumes 1 to 42 1987 Lyon, France: International Agency for Research on Cancer 3482203

[B8] Oldreive C Rice-Evans C The mechanisms for nitration and nitrotyrosine formation in vitro and in vivo: impact of diet Free Radic Res 2001 35 3 215 231 10.1080/10715760100300761 11697121

[B9] Fitzsimons JT Orson NV el-Aaser AA Effects of soyabean and ascorbic acid on experimental carcinogenesis Comp Biochem Physiol A 1989 93 1 285 290 10.1016/0300-9629(89)90218-1 2568231

[B10] Mokhtar NM el-Aaser AA el-Bolkainy MN Ibrahim HA el-Din NB Moharram NZ Effect of soybean feeding on experimental carcinogenesis – III. Carcinogenecity of nitrite and dibutylamine in mice: a histopathological study Eur J Cancer Clin Oncol 1988 24 3 403 411 10.1016/S0277-5379(98)90009-8 3383943

[B11] Tannenbaum SR Mergens W Reaction of nitrite with vitamins C and E Ann N Y Acad Sci 1980 355 267 279 10.1111/j.1749-6632.1980.tb21345.x 6940481

[B12] Gonzalez CA Riboli E Diet and cancer prevention: where we are, where we are going Nutr Cancer 2006 56 2 225 231 10.1207/s15327914nc5602_14 17474869

[B13] Flood A Rastogi T Wirfalt E Mitrou PN Reedy J Subar AF Kipnis V Mouw T Hollenbeck AR Leitzmann M Dietary patterns as identified by factor analysis and colorectal cancer among middle-aged Americans Am J Clin Nutr 2008 88 1 176 184 18614739 10.1093/ajcn/88.1.176 PMC2495056

[B14] Nothlings U Schulze MB Weikert C Boeing H Schouw YT van der Bamia C Benetou V Lagiou P Krogh V Beulens JW Intake of vegetables, legumes, and fruit, and risk for all-cause, cardiovascular, and cancer mortality in a European diabetic population J Nutr 2008 138 4 775 781 18356334 10.1093/jn/138.4.775

[B15] Vainio H Weiderpass E Fruit and vegetables in cancer prevention Nutr Cancer 2006 54 1 111 142 10.1207/s15327914nc5401_13 16800779

[B16] World Cancer Res. Fund Panel. Food, nutrition, and the prevention of cancer: a global perspective 2006 Washington, DC: American Institute for Cancer Res

[B17] Sarasua S Savitz DA Cured and broiled meat consumption in relation to childhood cancer: Denver, Colorado (United States) Cancer Causes Control 1994 5 2 141 148 10.1007/BF01830260 8167261

[B18] Peters JM Preston-Martin S London SJ Bowman JD Buckley JD Thomas DC Processed meats and risk of childhood leukemia (California, USA) Cancer Causes Control 1994 5 2 195 202 10.1007/BF01830266 8167267

[B19] Kwan ML Block G Selvin S Month S Buffler PA Food consumption by children and the risk of childhood acute leukemia Am J Epidemiol 2004 160 11 1098 1107 10.1093/aje/kwh317 15561989

[B20] Liu CY Hsu YH Pan PC Wu MT Ho CK Su L Xu X Li Y Christiani DC Maternal and offspring genetic variants of AKR1C3 and the risk of childhood leukemia Carcinogenesis 2008 29 5 984 990 10.1093/carcin/bgn071 18339682 PMC2902386

[B21] Yu CL Wang SF Pan PC Wu MT Ho CK Smith TJ Li Y Pothier L Christiani DC Residential exposure to petrochemicals and the risk of leukemia: using geographic information system tools to estimate individual-level residential exposure Am J Epidemiol 2006 164 3 200 207 10.1093/aje/kwj182 16754633

[B22] Olshan AF Smith J Cook MN Grufferman S Pollock BH Stram DO Seeger RC Look AT Cohn SL Castleberry RP Hormone and fertility drug use and the risk of neuroblastoma: a report from the Children's Cancer Group and the Pediatric Oncology Group Am J Epidemiol 1999 150 9 930 938 10547138 10.1093/oxfordjournals.aje.a010101

[B23] Ross JA Dietary flavonoids and the MLL gene: A pathway to infant leukemia? Proc Natl Acad Sci USA 2000 97 9 4411 4413 10781030 10.1073/pnas.97.9.4411 PMC34309

[B24] Tzeng WY Pan WH National Nutrition and Health Survey in Taiwan, 1993–1996 (In Chinese) NAHSIT

[B25] Steinmetz KA Potter JD Vegetables, fruit, and cancer. II. Mechanisms Cancer Causes Control 1991 2 6 427 442 10.1007/BF00054304 1764568

[B26] Ross JA Kasum CM Davies SM Jacobs DR Folsom AR Potter JD Diet and risk of leukemia in the Iowa Women's Health Study Cancer Epidemiol Biomarkers Prev 2002 11 8 777 781 12163333

[B27] Kwiatkowski A Dietary and other environmental risk factors in acute leukaemias: a case-control study of 119 patients Eur J Cancer Prev 1993 2 2 139 146 10.1097/00008469-199303000-00006 8461864

[B28] Dai Q Franke AA Jin F Shu XO Hebert JR Custer LJ Cheng J Gao YT Zheng W Urinary excretion of phytoestrogens and risk of breast cancer among Chinese women in Shanghai Cancer Epidemiol Biomarkers Prev 2002 11 9 815 821 12223424

[B29] Wu AH Ziegler RG Horn-Ross PL Nomura AM West DW Kolonel LN Rosenthal JF Hoover RN Pike MC Tofu and risk of breast cancer in Asian-Americans Cancer Epidemiol Biomarkers Prev 1996 5 11 901 906 8922298

[B30] Jacobsen BK Knutsen SF Fraser GE Does high soy milk intake reduce prostate cancer incidence? The Adventist Health Study (United States) Cancer Causes Control 1998 9 6 553 557 10.1023/A:1008819500080 10189040

[B31] Lee MM Gomez SL Chang JS Wey M Wang RT Hsing AW Soy and isoflavone consumption in relation to prostate cancer risk in China Cancer Epidemiol Biomarkers Prev 2003 12 7 665 668 12869409

[B32] Wu AH Yang D Pike MC A meta-analysis of soyfoods and risk of stomach cancer: the problem of potential confounders Cancer Epidemiol Biomarkers Prev 2000 9 10 1051 1058 11045787

[B33] Ward MH Pan WH Cheng YJ Li FH Brinton LA Chen CJ Hsu MM Chen IH Levine PH Yang CS Dietary exposure to nitrite and nitrosamines and risk of nasopharyngeal carcinoma in Taiwan Int J Cancer 2000 86 5 603 609 10.1002/(SICI)1097-0215(20000601)86:5<603::AID-IJC1>3.0.CO;2-H 10797279

[B34] Anderson JW Smith BM Washnock CS Cardiovascular and renal benefits of dry bean and soybean intake Am J Clin Nutr 1999 70 3 Suppl 464S 474S 10479219 10.1093/ajcn/70.3.464s

[B35] Yamakoshi J Piskula MK Izumi T Tobe K Saito M Kataoka S Obata A Kikuchi M Isoflavone aglycone-rich extract without soy protein attenuates atherosclerosis development in cholesterol-fed rabbits J Nutr 2000 130 8 1887 1893 10917898 10.1093/jn/130.8.1887

[B36] Chen C Kong AN Dietary chemopreventive compounds and ARE/EpRE signaling Free Radic Biol Med 2004 36 12 1505 1516 10.1016/j.freeradbiomed.2004.03.015 15182853

[B37] Lee YY Westphal AH de Haan LH Aarts JM Rietjens IM van Berkel WJ Human NAD(P)H:quinone oxidoreductase inhibition by flavonoids in living cells Free Radic Biol Med 2005 39 2 257 265 10.1016/j.freeradbiomed.2005.03.013 15964517

[B38] Messina M Barnes S The role of soy products in reducing risk of cancer J Natl Cancer Inst 1991 83 8 541 546 10.1093/jnci/83.8.541 1672382

[B39] Fotsis T Pepper M Adlercreutz H Hase T Montesano R Schweigerer L Genistein, a dietary ingested isoflavonoid, inhibits cell proliferation and in vitro angiogenesis J Nutr 1995 125 3 Suppl 790S 797S 7533831 10.1093/jn/125.suppl_3.790S

[B40] Markovits J Linassier C Fosse P Couprie J Pierre J Jacquemin-Sablon A Saucier JM Le Pecq JB Larsen AK Inhibitory effects of the tyrosine kinase inhibitor genistein on mammalian DNA topoisomerase II Cancer Res 1989 49 18 5111 5117 2548712

[B41] Akiyama T Ishida J Nakagawa S Ogawara H Watanabe S Itoh N Shibuya M Fukami Y Genistein, a specific inhibitor of tyrosine-specific protein kinases J Biol Chem 1987 262 12 5592 5595 3106339

[B42] Gale KB Ford AM Repp R Borkhardt A Keller C Eden OB Greaves MF Backtracking leukemia to birth: identification of clonotypic gene fusion sequences in neonatal blood spots Proc Natl Acad Sci USA 1997 94 25 13950 13954 9391133 10.1073/pnas.94.25.13950 PMC28413

[B43] Ford AM Ridge SA Cabrera ME Mahmoud H Steel CM Chan LC Greaves M In utero rearrangements in the trithorax-related oncogene in infant leukaemias Nature 1993 363 6427 358 360 10.1038/363358a0 8497319

[B44] Ford AM Pombo-de-Oliveira MS McCarthy KP MacLean JM Carrico KC Vincent RF Greaves M Monoclonal origin of concordant T-cell malignancy in identical twins Blood 1997 89 1 281 285 8978302

[B45] Thompson JR Gerald PF Willoughby ML Armstrong BK Maternal folate supplementation in pregnancy and protection against acute lymphoblastic leukaemia in childhood: a case-control study Lancet 2001 358 9297 1935 1940 10.1016/S0140-6736(01)06959-8 11747917

[B46] Jensen CD Block G Buffler P Ma X Selvin S Month S Maternal dietary risk factors in childhood acute lymphoblastic leukemia (United States) Cancer Causes Control 2004 15 6 559 570 10.1023/B:CACO.0000036161.98734.17 15280635

[B47] Hassan HT Ajoene (natural garlic compound): a new anti-leukaemia agent for AML therapy Leuk Res 2004 28 7 667 671 10.1016/j.leukres.2003.10.008 15158086

[B48] Michels KB The role of nutrition in cancer development and prevention Int J Cancer 2005 114 2 163 165 10.1002/ijc.20662 15540221

